# Practical and clinical utility of non-invasive vagus nerve stimulation (nVNS) for the acute treatment of migraine: a post hoc analysis of the randomized, sham-controlled, double-blind PRESTO trial

**DOI:** 10.1186/s10194-018-0928-1

**Published:** 2018-10-19

**Authors:** Licia Grazzi, Cristina Tassorelli, Marina de Tommaso, Giulia Pierangeli, Paolo Martelletti, Innocenzo Rainero, Pierangelo Geppetti, Anna Ambrosini, Paola Sarchielli, Eric Liebler, Piero Barbanti, on Behalf of the PRESTO Study Group, Cristina Tassorelli, Vito Bitetto, Roberto De Icco, Daniele Martinelli, Grazia Sances, Monica Bianchi, Licia Grazzi, Anna Maria Padovan, Marina de Tommaso, Katia Ricci, Eleonora Vecchio, Pietro Cortelli, Sabina Cevoli, Giulia Pierangeli, Rossana Terlizzi, Paolo Martelletti, Andrea Negro, Gabriella Addolorata Chiariello, Innocenzo Rainero, Paola De Martino, Annalisa Gai, Flora Govone, Federica Masuzzo, Elisa Rubino, Maria Claudia Torrieri, Alessandro Vacca, Pierangelo Geppetti, Alberto Chiarugi, Francesco De Cesaris, Simone Li Puma, Chiara Lupi, Ilaria Marone, Anna Ambrosini, Armando Perrotta, Paola Sarchielli, Laura Bernetti, Ilenia Corbelli, Michele Romoli, Simone Simoni, Angela Verzina, Piero Barbanti, Cinzia Aurilia, Gabriella Egeo, Luisa Fofi, Eric Liebler, Annelie Andersson, Lia Spitzer, Juana Marin, Candace McClure, Lisa Thackeray, Maria Giovanna Baldi, Daniela Di Maro

**Affiliations:** 1 Neuroalgology Unit, Carlo Besta Neurological Institute and Foundation, Milan, Italy; 2 Headache Science Centre, IRCCS C. Mondino Foundation, Pavia, Italy; 3 Department of Brain and Behavioral Sciences, University of Pavia, Pavia, Italy; 4 Neurophysiology and Pain Unit, University of Bari Aldo Moro, Bari, Italy; 5 IRCCS Istituto delle Scienze Neurologiche di Bologna, Bologna, Italy; 6 Department of Clinical and Molecular Medicine, Sapienza University, Rome, Italy; 7 Department of Neuroscience, University of Turin, Turin, Italy; 8 Headache Centre, University Hospital of Careggi, Florence, Italy; 9 IRCCS Neuromed, Pozzilli (IS), Italy; 10 Neurologic Clinic, Santa Maria della Misericordia Hospital, Perugia, Italy; 11 electroCore, Inc, Basking Ridge, NJ USA; 12 Headache and Pain Unit, IRCCS San Raffaele Pisana, Rome, Italy; 13 Department of Fondazione IRCCS Istituto Neurologico C. Besta, U.O. Neurologia III – Cefalee e Neuroalgologia, Via Celoria 11, 20133 Milan, Italy

**Keywords:** Neuromodulation, Vagus nerve stimulation, Post hoc analysis, Migraine, Rescue medication, Pain intensity

## Abstract

**Background:**

The PRESTO study of non-invasive vagus nerve stimulation (nVNS; gammaCore®) featured key primary and secondary end points recommended by the International Headache Society to provide Class I evidence that for patients with an episodic migraine, nVNS significantly increases the probability of having mild pain or being pain-free 2 h post stimulation. Here, we examined additional data from PRESTO to provide further insights into the practical utility of nVNS by evaluating its ability to consistently deliver clinically meaningful improvements in pain intensity while reducing the need for rescue medication.

**Methods:**

Patients recorded pain intensity for treated migraine attacks on a 4-point scale. Data were examined to compare nVNS and sham with regard to the percentage of patients who benefited by at least 1 point in pain intensity. We also assessed the percentage of attacks that required rescue medication and pain-free rates stratified by pain intensity at treatment initiation.

**Results:**

A significantly higher percentage of patients who used acute nVNS treatment (*n* = 120) vs sham (*n* = 123) reported a ≥ 1-point decrease in pain intensity at 30 min (nVNS, 32.2%; sham, 18.5%; *P* = 0.020), 60 min (nVNS, 38.8%; sham, 24.0%; *P* = 0.017), and 120 min (nVNS, 46.8%; sham, 26.2%; *P* = 0.002) after the first attack. Similar significant results were seen when assessing the benefit in all attacks. The proportion of patients who did not require rescue medication was significantly higher with nVNS than with sham for the first attack (nVNS, 59.3%; sham, 41.9%; *P* = 0.013) and all attacks (nVNS, 52.3%; sham, 37.3%; *P* = 0.008). When initial pain intensity was mild, the percentage of patients with no pain after treatment was significantly higher with nVNS than with sham at 60 min (all attacks: nVNS, 37.0%; sham, 21.2%; *P* = 0.025) and 120 min (first attack: nVNS, 50.0%; sham, 25.0%; *P* = 0.018; all attacks: nVNS, 46.7%; sham, 30.1%; *P* = 0.037).

**Conclusions:**

This post hoc analysis demonstrated that acute nVNS treatment quickly and consistently reduced pain intensity while decreasing rescue medication use. These clinical benefits provide guidance in the optimal use of nVNS in everyday practice, which can potentially reduce use of acute pharmacologic medications and their associated adverse events.

**Trial registration:**

ClinicalTrials.gov identifier: NCT02686034.

## Background

**Fig. 1:**
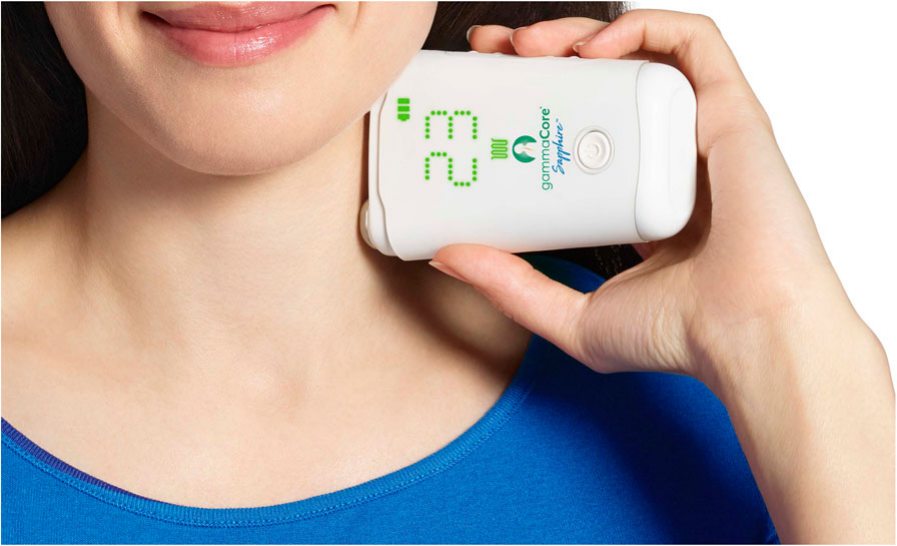
The Non-invasive Vagus Nerve Stimulation Device. Note: A previous model of the nVNS device was used by patients in the PRospectivE Study of nVNS for the acute Treatment Of migraine (PRESTO) trial. Image provided courtesy of electroCore, Inc. Abbreviation: nVNS, non-invasive vagus nerve stimulation

Non-invasive vagus nerve stimulation (nVNS; gammaCore®; electroCore, Inc., Basking Ridge, NJ, USA) (Fig. 1) is a safe and effective treatment for patients with migraine [1–4]. The therapy is practical, flexible, and easy to use, with the lack of drug-drug interactions allowing its use as a complement to existing treatments [5, 6]. nVNS reduces the need for pharmacologic therapies and their related side effects in the treatment of migraine [7, 8]. In the PRospectivE Study of nVNS for the acute Treatment Of migraine (PRESTO), nVNS was superior to sham for the majority of end points including pain freedom, pain relief, and ≥ 50% responder rates at various time points [4]. Adverse events were minimal and mostly mild in severity [4]. The PRESTO trial provided Class I evidence that for patients with an episodic migraine, nVNS significantly increases the probability of having mild pain or being pain-free 2 h post stimulation.

In this post hoc analysis, we provide further insight into the practical utility of acute nVNS treatment through the reporting of clinically relevant end points that extend beyond the traditional key end points recommended for pivotal clinical trials by the International Headache Society (IHS) [9]. The objectives of this analysis were to evaluate the likelihood of experiencing at least a 1-point decrease in pain intensity while reducing the need for rescue medication and to assess whether treatment of a migraine attack when the pain is mild affects the efficacy of nVNS.

## Methods

### Study design

The methods for the prospective, double-blind, randomized, sham-controlled, multicenter PRESTO study were previously reported (ClinicalTrials.gov identifier: NCT02686034) [4]. The study took place at 10 Italian sites from January 11, 2016, through March 31, 2017, and consisted of three 4-week periods: 1) run-in, 2) double-blind, and 3) open-label periods. During the run-in period, patients received their standard medications. In the double-blind period, patients were randomly assigned to either nVNS or sham treatment. During the open-label period, all patients received nVNS treatment. Patients were instructed to treat up to 5 migraine attacks with nVNS or sham during the double-blind period and up to 5 additional attacks with nVNS during the open-label period. Only one attack could be treated within a 48-h period.

### Study population

Patients were 18 to 75 years of age and had a previous diagnosis of migraine with or without aura according to the *International Classification of Headache Disorders, 3rd edition* criteria (*ICHD-3*) [10]. Key exclusion criteria included history of secondary headache, another significant pain disorder, uncontrolled hypertension, botulinum toxin injections in the last 6 months, and head or neck nerve blocks in the last 2 months.

### Intervention

**Fig. 2:**
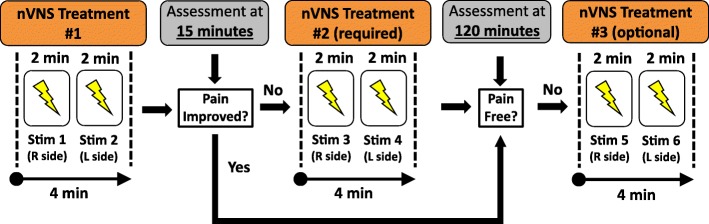
PRESTO Treatment Paradigm. Abbreviations: L, left; nVNS, non-invasive vagus nerve stimulation; R, right; Stim, stimulation

Within 20 min of migraine pain onset, patients self-administered two bilateral 120-s stimulations (ie, 1 stimulation each to the right and left cervical branch of the vagus nerve) (Fig. 2). If pain did not decrease 15 min after nVNS administration, the bilateral stimulations were repeated. At 120 min, an optional additional set of stimulations was repeated if the patient was not pain-free, and optional rescue medication could be used. Any rescue medication use before the 120-min assessment was considered treatment failure. Patients maintained preventive migraine medication use at a stable dose and frequency during the 2 months before enrollment and throughout the study. No new preventive medication was permitted during the study.

### End points

The percentage of patients with a ≥ 1-point reduction in pain intensity on a 4-point scale (0, *no pain*; 1, *mild pain*; 2, *moderate pain*; 3, *severe pain*) was measured at 30, 60, and 120 min after the first treated attack of the double-blind and open-label periods. Rescue medication use and pain-free rates at 30, 60, and 120 min stratified by initial pain intensity were evaluated for the first treated attack of both periods. Similar analyses were performed for all attacks for both periods.

### Statistical analyses

All analyses were evaluated in the *intent-to-treat (ITT) population*, defined as patients who treated at least one migraine attack in the double-blind period. Proportions of patients with pain reductions of ≥1 point and proportions of patients who did not use rescue medication were estimated for the first attack using logistic regression models adjusted for baseline pain score, preventive medication use, and presence of aura. *P*-values for comparisons between the nVNS and sham groups were from the covariate-adjusted logistic regression models. Pain-free rates for the first attack were presented as proportion and 95% exact binomial confidence interval (CI). *P*-values for comparison of pain-free rates for the first attack between the nVNS and sham groups in the double-blind period were from the chi-square test or Fisher exact test, as appropriate. To estimate proportions of all attacks that achieved ≥1-point pain reductions and proportions of all attacks not requiring rescue medication, generalized linear mixed-effects regression models adjusted for baseline pain score, preventive medication use, and presence of aura were used. Odds ratios and 95% CIs for comparisons of rates between the nVNS and sham groups for all attacks were from the covariate-adjusted generalized linear mixed-effects regression models; *P*-values were from resulting F tests. To estimate pain-free rates for all attacks, unadjusted generalized linear mixed-effects regression models were used; *P*-values comparing nVNS with sham were from resulting F tests. All data were analyzed using SAS® 9.4 (SAS Institute Inc., Cary, NC, USA).

## Results

### Patients

Full details on patient disposition, demographics, and baseline characteristics in the PRESTO study were reported previously [4]. A total of 285 patients with episodic migraine were enrolled, with 248 randomly assigned to the nVNS (*n* = 122) and sham (*n* = 126) groups. The ITT population consisted of 120 patients randomized to receive nVNS and 123 patients randomized to receive sham. Patients were < 50 years of age at migraine onset, with a frequency of 3 to 8 attacks per month. Demographic and baseline characteristics were similar between the nVNS and sham groups. More patients in the nVNS group than in the sham group initiated treatment when attack intensity was severe (first attack: nVNS, 23.5%; sham, 15.1%; all attacks: nVNS, 25.1%; sham, 17.6%). A total of 238 patients (nVNS, *n* = 117; sham, *n* = 121) completed the open-label period.

### ≥1-point reduction in pain intensity

**Fig. 3:**
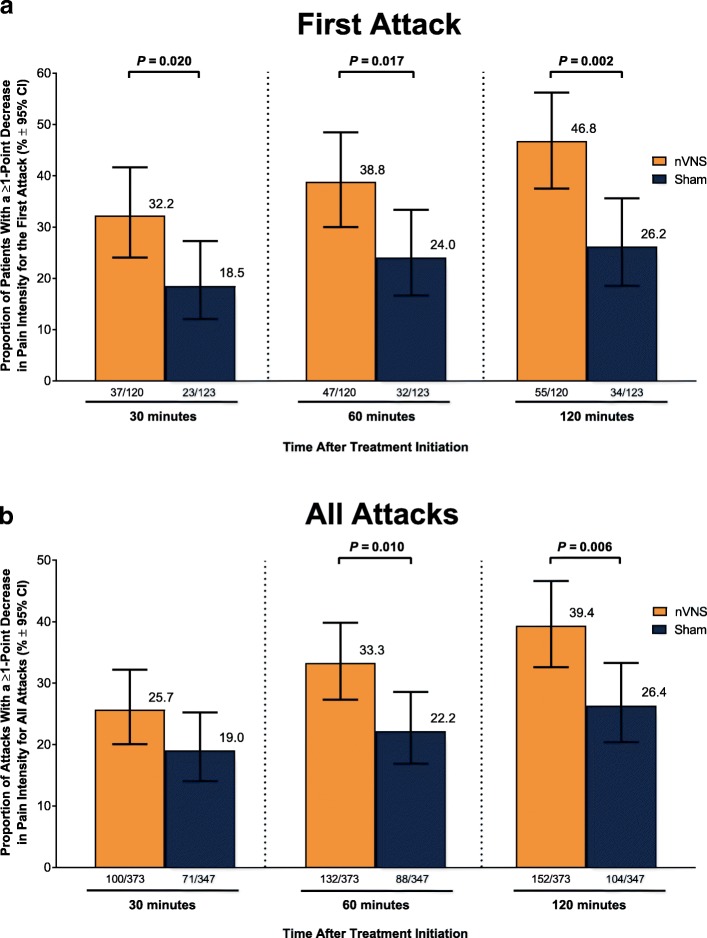
≥1-Point Reduction in Pain Intensity at 30, 60, and 120 Minutes for (**a**) First Attack and (**b**) All Attacks. Models are adjusted for the patients' baseline pain score, use of preventive therapies, and presence of aura; data for number of patients are unadjusted numbers. Abbreviation: nVNS, non-invasive vagus nerve stimulation

Acute nVNS treatment provided clinically meaningful and significant benefits vs sham in the double-blind period. For the first treated attack (Fig. 3A), percentages of patients who recorded a ≥ 1-point reduction in pain intensity were significantly greater in the nVNS group than in the sham group at 30 min (nVNS, 32.2%; sham, 18.5%; *P* = 0.020), 60 min (nVNS, 38.8%; sham, 24.0%; *P* = 0.017), and 120 min (nVNS, 46.8%; sham, 26.2%; *P* = 0.002). For all treated attacks (Fig. 3B), significantly more ≥1-point pain improvements were seen with nVNS than with sham at 60 min (nVNS, 33.3%; sham, 22.2%; *P* = 0.010) and 120 min (nVNS, 39.4%; sham, 26.4%; *P* = 0.006).

### Percentage of patients not requiring rescue medication

**Fig. 4:**
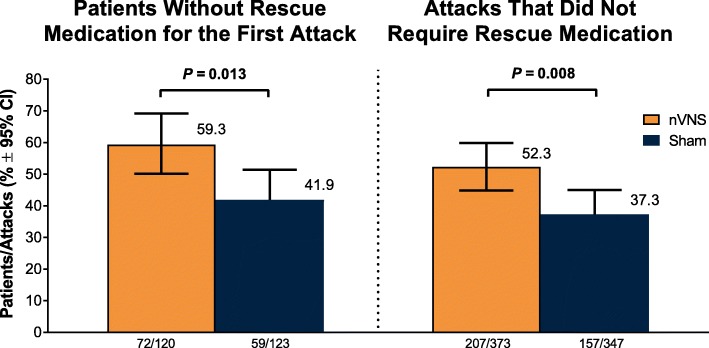
Rescue Medication Use. Models are adjusted for the patients’ baseline pain score, use of preventive therapies, and presence of aura; data for number of patients are unadjusted numbers. Abbreviation: nVNS, non-invasive vagus nerve stimulation

The proportion of patients who did not use rescue medication was significantly higher with nVNS than with sham for the first attack (nVNS, 59.3%; sham, 41.9%; *P* = 0.013) and for all attacks (nVNS, 52.3%; sham, 37.3%; *P* = 0.008) (Fig. 4).

### Pain-free rates by initial pain intensity levels

**Fig. 5:**
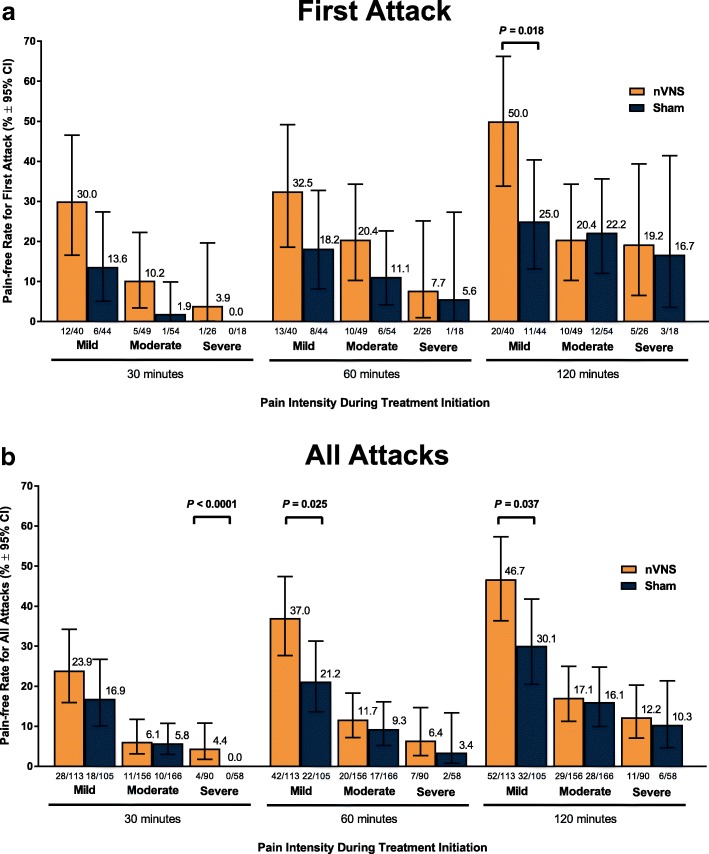
Pain-free Rates at 30, 60, and 120 Minutes for (**a**) First Attack and (**b**) All Attacks. Abbreviation: nVNS, non-invasive vagus nerve stimulation

Differences in pain-free rates between nVNS and sham were more pronounced in patients who initiated treatment when their attack was mild than for those who waited until the pain was moderate or severe to treat their attack. The percentage of patients who successfully aborted a mild first migraine attack was significantly higher with nVNS than with sham at 120 min (nVNS, 50.0%; sham, 25.0%; *P* = 0.018) (Fig. 5A). When all mild attacks were considered, the percentages that became pain-free remained significantly higher with nVNS than with sham at 60 min (nVNS, 37.0%; sham, 21.2%; *P* = 0.025) and at 120 min (nVNS, 46.7%; sham, 30.1%; *P* = 0.037) (Fig. 5B). When the initial pain was severe, the percentage of all treated attacks that were aborted was significantly higher with nVNS than with sham at 30 min (nVNS, 4.4%; sham, 0.0%; *P* < 0.0001) (Fig. 5B). The statistical benefit of nVNS vs sham in treating these severe attacks at 30 min may not be reliable given that the sham group had a proportion size of zero. When the initial pain intensity was moderate or severe, pain-free rates were not significantly different between the nVNS group and the sham group, though they were generally higher in the nVNS group at all time points.

### Clinical utility outcomes in the open-label period

Therapeutic benefits observed in the nVNS group at 120 min during the double-blind period (ie, ≥1-point reductions in pain, decreases in rescue medication use, and improvements in pain-free rates by initial pain level) were sustained in the 4-week open-label period during which all patients received nVNS (Table 1).

**Table 1:** Summary of Clinical Utility Outcomes at 120 Minutes After Treatment Initiation (Double-blind and Open-label Periods)

End Point	Pain Intensity at Treatment Initiation	Double-blind Period		Open-label Period
		nVNS	Sham	nVNS
First Attack				
% ≥1-point pain improvement	–	46.8**	26.2	42.9
% without rescue medication	–	59.3*	41.9	49.1
% pain-free response	Mild	50.0*	25.0	56.9
	Moderate	20.4	22.2	14.8
	Severe	19.2	16.7	23.3
All Attacks				
% ≥1-point pain improvement	–	39.4**	26.4	41.8
% without rescue medication	–	52.3**	37.3	49.7
% pain-free response	Mild	46.7*	30.1	48.6
	Moderate	17.1	16.1	13.8
	Severe	12.2	10.3	14.6

*P < 0.05 vs sham in the double-blind period

**P < 0.01 vs sham in the double-blind period

*Abbreviation*: *nVNS* non-invasive vagus nerve stimulation

## Discussion

Treatment with nVNS consistently led to clinically relevant reductions in pain while reducing the need for rescue medication for the first and all attacks. The ability of nVNS to offer measurable pain relief for patients without increasing their exposure to pharmacologic adverse events or medication overuse provides a practical rationale for its early use as an acute treatment [11]. Patients who initiated their treatment when their migraine was mild were more likely to abort their attacks than those who treated when their pain was more severe, a finding consistent with clinical studies that demonstrated the efficacy of pharmacologic therapies during early stages of migraine attacks [12, 13] and when pain was still mild [14–18]. The combination of efficacy and tolerability with nVNS might provide patients with the confidence to initiate treatment earlier in their attacks compared with pharmacologic options that, in clinical experience, are often initiated when the pain is more severe because of concerns with drug availability, overuse, and adverse events [14, 19–21].

Findings from mechanistic studies further support the initiation of nVNS treatment as early as possible to facilitate greater reductions in pain [22, 23]. Early nVNS treatment may reduce central excitability by blunting subsequent neurotransmitter release associated with severe migraine pain [23]. Two additional animal models demonstrated that nVNS inhibited expression of proteins associated with central sensitization of trigeminal neurons and reduced susceptibility to cortical spreading depression [24, 25]. These findings provide the mechanistic rationale for optimizing treatment response with early nVNS administration, before these neurophysiological activities are established.

Migraine may share common mechanistic pathways and latent causes with comorbid pain disorders such as fibromyalgia, chronic pelvic pain, and myofascial pain syndromes [26, 27]. Consistent with IHS recommendations, the PRESTO study excluded patients with such disorders although they are frequently seen in clinical practice [4, 9, 26]. Improvements in fibromyalgia symptoms have been observed in patients receiving pharmacologic migraine medication [26], suggesting that other effective migraine therapies such as nVNS could also have expanded benefits for these difficult-to-treat patients. In mechanistic studies, nVNS was shown to suppress pain markers that are not necessarily specific to migraine [22–24, 28, 29]. This potential broader effect on pain is further supported by a proof-of-concept study of adjunctive implantable VNS for fibromyalgia, which demonstrated improvement in pain, overall wellness, and physical function for 5 patients implanted with VNS [30]. These mechanistic and clinical insights suggest that evaluation of nVNS as a possible treatment for patients with migraine and comorbidities such as fibromyalgia is warranted.

Post hoc analyses of clinical trials can be criticized for the use of less-rigorous methods or nonrepresentative subsets of a larger population [31–33]. We performed these post hoc analyses on the entire study population, which allowed the identification of a subpopulation of subjects who were more likely to respond to nVNS (ie, those who treat the attack when pain is mild). The findings are further strengthened by the analysis of data collected during the observational open-label phase [11]. There are currently no guidelines on the study design of neuromodulation devices in migraine, and well-controlled studies must rely only on the recommendations of the IHS for controlled trials of drugs [9]. These pharmacologic guidelines may be suboptimal for studies of neuromodulation devices because of the differing mechanisms of action and interventional targets. Our findings suggest that rigorous post hoc analyses of well-controlled clinical trials could inform future guidelines for neuromodulation devices. Although the end points in this analysis are not the previously reported primary or key secondary end points recommended by the IHS, they provide additional insight into the practical clinical utility of nVNS in relieving pain while reducing rescue medication use.

A limiting factor of the PRESTO trial was that the sham device, which delivered an appreciable electrical signal, appears to have had some level of vagal activation [34]. The design of sham devices for neuromodulation studies is inherently difficult because a compromise must be reached between maintaining blinding with a noticeable stimulation and minimizing an active effect. A sham device that produces an active signal could obscure the actual effects of the verum device, thus reducing the opportunity to demonstrate therapeutic benefits above that of the sham device. We believe that the sham signal in the PRESTO study likely provided a detectable degree of active treatment effects that potentially masked some of the differences between the nVNS and sham groups in the current analysis [34].

nVNS is a practical treatment option with considerable clinical utility in the acute treatment of migraine. The likelihood that nVNS will quickly reduce pain by at least 1 point and decrease the use of rescue medication highlights its favorable risk-benefit profile [1–3, 9]. Significant reductions in the use of acute pharmacologic therapies for the nVNS group in this analysis may encourage patient confidence and adherence to nVNS as it provides an opportunity to minimize or avoid the potential limitations associated with traditional acute migraine medications, including drug-drug interactions, pharmacologic adverse events, and medication overuse [35–37]. nVNS offers flexibility, efficacy, and established safety and tolerability that may encourage earlier use than is typically seen with conventional acute therapies. Although demonstrated to be beneficial, pharmacologic treatments are often reserved for pain that is more severe because of a range of issues, including medication-related tolerability and a potentially insufficient availability of other acute medications [4, 38]. This analysis supports nVNS as a practical and effective alternative that can be used frequently and as early in an attack as desired to decrease migraine pain while reducing the need for rescue medication and minimizing drug-related adverse events.

## Conclusions

These data highlight clinically important benefits of nVNS as an acute treatment of migraine. nVNS decreased pain by at least 1 point while reducing rescue medication use in most migraine attacks. Unlike most pharmacologic options, nVNS has the flexibility to be used alone or as adjunctive therapy for multiple attacks without risk of pharmacologic interactions and adverse events. These benefits, along with its convenience and ease of use, make nVNS an appealing practical option for the acute treatment of migraine.

## Abbreviations

CI: confidence interval
ICHD-3: International Classification of Headache Disorders, 3rd edition
IHS: International Headache Society
ITT: intent-to-treat
L: left
nVNS: non-invasive vagus nerve stimulation
PRESTO: Prospective study of nVNS for the acute treatment of migraine
R: right
Stim: stimulation
