# Correction to: Practical and clinical utility of non-invasive vagus nerve stimulation (nVNS) for the acute treatment of migraine: a post hoc analysis of the randomized, sham-controlled, double-blind PRESTO trial

**DOI:** 10.1186/s10194-018-0948-x

**Published:** 2019-01-07

**Authors:** Licia Grazzi, Cristina Tassorelli, Marina de Tommaso, Giulia Pierangeli, Paolo Martelletti, Innocenzo Rainero, Pierangelo Geppetti, Anna Ambrosini, Paola Sarchielli, Eric Liebler, Piero Barbanti, on Behalf of the PRESTO Study Group

**Affiliations:** 1 Neuroalgology Unit, Carlo Besta Neurological Institute and Foundation, Milan, Italy; 2 Headache Science Centre, IRCCS C. Mondino Foundation, Pavia, Italy; 3 Department of Brain and Behavioral Sciences, University of Pavia, Pavia, Italy; 4 Neurophysiology and Pain Unit, University of Bari Aldo Moro, Bari, Italy; 5 IRCCS Istituto delle Scienze Neurologiche di Bologna, Bologna, Italy; 6 Department of Clinical and Molecular Medicine, Sapienza University, Rome, Italy; 7 Department of Neuroscience, University of Turin, Turin, Italy; 8 Headache Centre, University Hospital of Careggi, Florence, Italy; 9 IRCCS Neuromed, Pozzilli (IS), Italy; 10 Neurologic Clinic, Santa Maria della Misericordia Hospital, Perugia, Italy; 11 electroCore, Inc, Basking Ridge, NJ USA; 12 Headache and Pain Unit, IRCCS San Raffaele Pisana, Rome, Italy; 13 Department of Fondazione IRCCS Istituto Neurologico C. Besta, U.O. Neurologia III – Cefalee e Neuroalgologia, Via Celoria 11, 20133 Milan, Italy

**Correction to: J Headache Pain (2018) 19:98**

**https://doi.org/10.1186/s10194-018-0928-1**

Following publication of the original article [1], the authors notified us that a part of the Figure 3 legend was omitted during the proofing stage.

The original publication has been corrected. The incorrect and correct figure legends are presented below.

• Originally published legend for Figure 3:

≥1-Point Reduction in Pain Intensity at 30, 60, and 120 Minutes for (a) First Attack and (b) All Attacks

• Corrected Figure 3 legend:

≥1-Point Reduction in Pain Intensity at 30, 60, and 120 Minutes for (a) First Attack and (b) All Attacks. Models are adjusted for the patients’ baseline pain score, use of preventive therapies, and presence of aura; data for number of patients are unadjusted numbers. Abbreviation: nVNS, non-invasive vagus nerve stimulation
